# Somatosensory abnormalities after infection with SARS-CoV-2 – A prospective case-control study in children and adolescents

**DOI:** 10.3389/fped.2022.977827

**Published:** 2022-10-03

**Authors:** Lynn Eitner, Christoph Maier, Folke Brinkmann, Anne Schlegtendal, Leona Knoke, Elena Enax-Krumova, Thomas Lücke

**Affiliations:** ^1^Department of Neuropediatrics, University Children’s Hospital, Ruhr University Bochum, Bochum, Germany; ^2^University Children’s Hospital, Ruhr University Bochum, Bochum, Germany; ^3^Department of Neurology, BG University Hospital Bergmannsheil gGmbH, Ruhr-University Bochum, Bochum, Germany

**Keywords:** COVID-19, SARS-CoV-2, quantitative sensory testing, somatosensory function, children, adolescents

## Abstract

**Background:**

Long-term neurological complaints after SARS-CoV-2 infection occur in 4–66% of children and adolescents. Controlled studies on the integrity of the peripheral nerve system are scarce. Therefore, we examined the somatosensory function in children and adolescents after SARS-CoV-2 infection in a case-control study compared with age-matched individuals.

**Materials and Methods:**

Eighty-one subjects after SARS-CoV-2 infection (*n* = 44 female, 11.4 ± 3.5 years, *n* = 75 SARS-CoV-2 seropositive, *n* = 6 PCR positive during infection and SARS-CoV-2 seronegative at the time point of study inclusion, *n* = 47 asymptomatic infection) were compared to 38 controls without SARS-CoV-2 infection (26 female, 10.3 ± 3.4 years, *n* = 15 with other infection within last 6 months). After standardised interviews and neurological examinations, large fibre (tactile and vibration detection thresholds) and small fibre (cold and warm detection thresholds, paradoxical heat sensation) functions were assessed on both feet following a validated protocol. After z-transformation of all values, all participants were compared to published reference values regarding the number of abnormal results. Additionally, the mean for all sensory parameters values of both study groups were compared to an ideal healthy population (with *z*-value 0 ± 1), as well as with each other, as previously described. Statistical analyses: *t*-test, Chi-squared test, and binominal test.

**Findings:**

None of the controls, but 27 of the 81 patients (33%, *p* < 0.001) reported persistent complaints 2.7 ± 1.9 (0.8–8.5) months after SARS-CoV-2 infection, most often reduced exercise capacity (16%), fatigue (13%), pain (9%), or paraesthesia (6%). Reflex deficits or paresis were missing, but somatosensory profiles showed significantly increased detection thresholds for thermal (especially warm) and vibration stimuli compared to controls. Approximately 36% of the patients after SARS-CoV-2, but none of the controls revealed an abnormal sensory loss in at least one parameter (*p* < 0.01). Sensory loss was characterised in 26% by large and 12% by small fibre dysfunction, the latter appearing more frequently in children with prior symptomatic SARS-CoV-2 infection. Myalgia/paraesthesia was indicative of somatosensory dysfunction. In all eight re-examined children, the nerve function recovered after 2–4 months.

**Interpretation:**

This study provides evidence that in a subgroup of children and adolescents previously infected with SARS-CoV-2, regardless of their complaints, the function of large or small nerve fibres is presumably reversibly impaired.

## Introduction

Persisting complaints >4 weeks after SARS-CoV-2 infection, have been reported in 4–66% of affected children and adolescents despite a mostly mild course of infection (1–3). However, most data are based only on patient-reported outcomes, and the underlying pathomechanisms of most reported complaints remain unclear, especially whether they are correlated to an actual organ damage. Remarkably, many children complaining of dyspnoea do not suffer from persistent deterioration of respiratory function (4).

Although neurological complications are very rare in children and adolescents, changes in both the central and peripheral nervous system have been described (5–8), similar to other viral respiratory infections (9, 10). Some of the reported complaints after SARS-CoV-2 infection might indicate an affection of the peripheral or central nervous system, for example, smell and taste disorder, persisting paraesthesia, myalgia, limb pain, and headache, which might be related to an affection of the somatosensory system (11). Different molecular mechanisms are discussed, that may be involved in the development of neurological complaints and pain (12). This seems to be a multifactorial pathophysiology. One possible explanation could be the influence of SARS-CoV-2 on the angiotensin-converting enzyme 2 and renin-angiotensin system (ACE2/RAS). SARS-CoV-2 binds to the ACE2 receptor, which is also expressed in neurons and glial cells and can thus cause damage to sensory neurons. In addition, there are processes such as macrophage activation and an abnormal production of cytokines as well as autoantibodies, which arise through molecular mimicry (12, 13). The cytokine storm during severe COVID-19 infection has also been discussed as a potential driving factor for the development of neuropathies and could contribute to the development of chronic pain after the acute infection has resolved (14). These processes can cause neuronal damage and thus lead to acute and chronic impairment of function or pain (12). Some case reports have descried transient sensory loss (hypoalgesia) in adults several months after COVID-19 (15). Preclinical models indicate that SARS-CoV-2 spike protein subverts pronociceptive signalling including vascular endothelial growth factor-A and neuropilin-1 receptor (16).

To the best of our knowledge, no studies have analysed the somatosensory function in children with and without persisting complaints after infection with SARS-CoV-2.

Therefore, we compared the somatosensory function between children and adolescents with and without previously confirmed SARS-CoV-2 infection in a case-control study and in relation to persistent complaints. We performed quantitative sensory testing (QST), a validated and non-invasive approach to investigate the somatosensory function, including both small and large nerve fibres and the corresponding central pathways (17–19), according to the internationally accepted protocol of the German Research Network for Neuropathic Pain (DFNS e.V.) (17, 20–22). Its advantages include established reference data for children and adolescents older than 6 years (23, 24) and previous validation as a tool to assess neuropathy symptoms in children, for example, with type I diabetes mellitus, cerebral palsy or survivors of paediatric acute lymphoblastic leukaemia (25–27). Second, we analysed the possible influence of age and severity of previous infection on somatosensory performance and the type of long-term complaints 2–4 months after infection.

## Materials and methods

The study was approved by the Ethical Committee of the Faculty of Medicine, Ruhr University Bochum, in July 2020 (Reg. no. 20-6927_1) and performed in accordance with the Declaration of Helsinki. Participants were recruited from August 2020 to May 2021.

### Participants

Children and adolescents (aged 6–18 years) suspected of having had SARS-CoV-2 infection and interested participants from a population-based cohort study of children screened for SARS-CoV-2 infection (CorKid – SARS-CoV-2 seroconversion in kids) were included in the study.

Participants had a history of asymptomatic or symptomatic infection [confirmed by a reverse transcriptase–polymerase chain reaction (PCR) and/or antinuclear SARS-CoV-2 IgM and IgG antibodies COI > 1.000]. In six cases of positive viral detection by PCR during the acute infection, no SARS-CoV-2 IgM and IgG antibodies were detectable at the time point of study inclusion. The control group consisted of children and adolescents without evidence of SARS-CoV-2 infection (negative PCR and antibodies) (Supplementary Table 1). Exclusion criteria in both groups were any neurological or psychiatric disorders, mental illnesses, or relevant chronic diseases. The pre-existing comorbidities listed in Supplementary Table 1 are bronchial asthma, respiratory allergy and atopic dermatitis, which have no effect on peripheral nerve fibre function. In one case, familial Mediterranean fever was present.

### Assessment procedure

A standardised interview included information about the date and severity of the prior infection, the results of the former PCR and/or SARS-CoV-2 antibody tests and a list of complaints (Table 1). The latter which were classified into four main categories: general complaints, neurological complaints, pain, and respiratory complaints. Participants with respiratory complaints such as dyspnoea underwent extensive lung function analyses, including body plethysmography and lung clearance index as recently described (4).

**TABLE 1:** Long-term complaints newly emerged after SARS-CoV-2 infection.

	Post-SARS-CoV-2-group with current complaints			
	
	All	Symptomatic infection (*n* = 35)	Asymptomatic infection (*n* = 47)	*P* value
**At least one complaint, No. (%)**	**27** (100)	**16** (46)	**11** (23)	**0.033**
**General complaints, No. (%)**	**19** (70)	**12** (75)	**7** (64)	**0.04**
Reduced physical capacity, No. (%)	13 (48)	9 (56)	4 (36)	0.035
Fatigue, No. (%)	11 (41)	7 (44)	4 (36)	0.13
Sleep disorder, No. (%)	6 (22)	3 (19)	3 (27)	0.71
Mental complaints, No. (%)	4 (15)	3 (19)	1 (9)	0.18
Cold feet, No. (%)	5 (19)	5 (31)	0 (0)	0.007
Skin alterations, No. (%)	1 (4)	1 (6)	0 (0)	0.030
**Potentially neurological symptoms, No. (%)**	**19** (70)	**12** (75)	**7** (64)	**0.04**
Smell/Taste dysfunction, No. (%)	9 (33)	6 (38)	3 (27)	0.12
Tingling paraesthesia, No. (%)	5 (19)	5 (31)	0 (0)	0.007
Vertigo, No. (%)	5 (19)	2 (13)	3 (27)	0.90
Muscle weakness, No. (%)	1 (4)	1 (6)	0 (0)	0.30
Dysphagia, No. (%)	1 (4)	1 (6)	0 (0)	0.30
**Pain, No. (%)**	**7** (26)	**5** (31)	**2** (18)	**0.11**
Headache, No. (%)	6 (22)	4 (25)	2 (18)	0.22
Myalgia, No. (%)	5 (19)	5 (31)	0 (0)	0.007
Joint pain, No. (%)	2 (7)	2 (13)	0 (0)	0.12
Burning pain, diffuse, No. (%)	1 (4)	1 (6)	0 (0)	0.30
**Respiratory complaints, No. (%)**	**12** (44)	**9** (56)	**3** (27)	**0.014**
Dyspnoea, No. (%)	9 (33)	7 (44)	2 (18)	0.024
Cough, No. (%)	3 (11)	2 (13)	1 (9)	0.39

Data are expressed as No. (%), unless specified otherwise. Bold values represent the categories of symptoms.

### Quantitative sensory testing

Quantitative sensory testing was performed by a trained examiner (LE) who was blinded to the SARS-CoV-2 status of the participants at the time of QST examination and analysis. Thermal and mechanical detection thresholds were assessed at both dorsal feet according to the internationally accepted and recommended standardised protocol of the German research Network on Neuropathic pain (DFNS) and included the following six parameters: cold and warm detection thresholds (CDT, WDT), thermal sensory limen (TSL), paradoxical heat sensations (PHS), tactile mechanical detection threshold (MDT), and vibration detection threshold (VDT) (22). All procedures have been described in detail recently (23, 28). CDT, WDT, and TSL were tested using the thermal sensory testing device Q-Sense (MEDOC, Israel) with a contact area of 16 mm × 16 mm and cut-off temperatures of 16 and 45°C. Participants pressed a button once they felt cold or warm to determine the CDT and WDT (means of three measurements, respectively). TSL was assessed during a series of six alternating warm and cold stimuli, and participants pressed a button once they felt a temperature change. PHS was assessed by asking whether children perceived cold stimuli as warm during TSL. MDT was assessed using a standardised set of von Frey-Hairs (Marstock nervtest, Optihair 2, 0.25–512 mN). The threshold was determined as the geometric mean of a series of measurements of five ascending and descending stimuli. For the VDT assessment, we used a Rydel-Seiffert tuning fork (128 Hz). VDT was defined as the mean of three measurements.

Geber et al. (29) were able to demonstrate good test-retest and interobserver reliability [*r* = 0.86 (TRR; range 0.67–0.93) and *r* = 0.83 (IO-R; range 0.56–0.89)] for this assessment in a multi-centre study. Even over a period of 4 months, very good long-term reliability (intraclass correlation coefficients: 0.68–0.90) could be demonstrated for the parameters used in this work (30).

### Statistics

All sensory parameters except PHS have been shown to be normally distributed (partly in log space) (28). Subsequently, the sensory results of all study participants were z-transformed (except PHS), with *z*-values < 0 representing loss of somatosensory function compared to the mean of the aged-matched reference dataset (23). Abnormal values are defined as values beyond the 95% confidence interval, i.e., *z*-values < −1.96 (indicating abnormal sensory loss, i.e., hypoesthesia) and *z*-values > 1.96 (indicating abnormal sensory gain, i.e., hyperaesthesia). The z-transformation normalises for age, gender, and tested body region, thus making the results comparable. PHS (absent in healthy individuals under normal conditions) is reported as original values as the arithmetic mean of occurrences of PHS (0–3). For each QST parameter, the value from the foot indicating a higher deviation from the cut-off value of the reference data (i.e., more sensory loss or more sensory gain, respectively) was used for further statistical analysis. QST results were defined as abnormal in case of any increased detection threshold, that is z-score of CDT, WDT, TSL, MDT, or VDT < −1.96, or if PHS was >0 (23).

Chi-squared tests were used to analyse the number of abnormally increased detections for each sensory parameter compared to the controls and to a theoretically assumed frequency of 2.5% for each loss and gain, resulting from the definition of abnormal values, which is outside the 95% confidence interval of healthy subjects. Binominal test was used to analyse the frequencies of persistent complaints because of the small number in the study population. Unpaired *t*-tests were used to compare each QST parameter with the control group, as described in previous studies (28, 31). Additionally, QST *z*-values were compared to the expected values of an ideal healthy population with a mean *z*-value = 0 and a standard deviation = 1.

## Results

### Patient cohort

**FIGURE 1:**
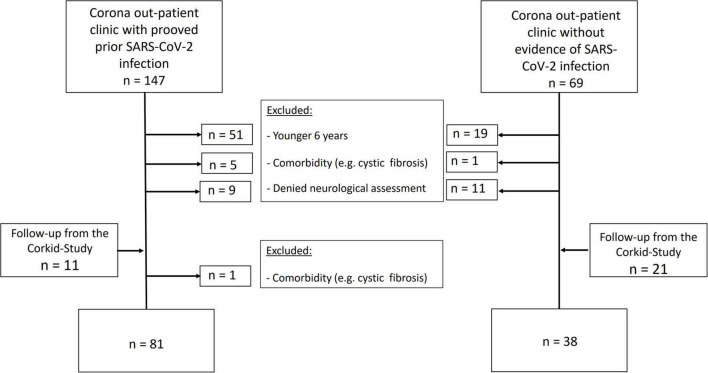
STROBE flowchart of included patients.

Study participants were recruited from 183 consecutive patients and their siblings from the Corona outpatient clinic and 32 subjects, who visited the CorKid study for follow-up (32) (for a STROBE-diagram, Figure 1). For the Corona outpatient clinic, families could call to register if any of their children had or were suspected of having a COVID-19 infection.

After exclusion due to age (<6 years), any comorbidity associated with peripheral neuropathy (like diabetic mellitus, cystic fibrosis) or declining neurological examinations, 81 children and adolescents with confirmed previous SARS-CoV-2 infection (age 11.4 ± 3.5 years, *n* = 67 tested with PCR, *n* = 14 tested with antibody test) were included (“SARS-CoV-2 group”). From the same cohort, 38 children and adolescent without history of previous SARS-CoV-2 infection and with negative antibody testing (age 10.3 ± 3.4 years) served as controls. Clinical and demographic data did not differ between the groups (Supplementary Table 1).

The assessment took place 2.8 ± 2.0 (0.8–8.8) months after PCR testing for SARS-CoV-2 or 2.6 ± 2.0 (0.7–6.3) months after symptoms of other viral infection. 42% of the participants in the SARS-CoV-2 group had a history of a symptomatic infection (*n* = 26 tested with PCR, *n* = 8 tested with antibody test) and 58% had asymptomatic infection (*n* = 41 tested with PCR, *n* = 6 tested with antibody test). In the control group, 40% reported other symptomatic viral infections in the 6 months prior to assessment. Symptoms during acute viral infections such as headache (24 vs. 11%), fever (25 vs. 13%), taste dysfunction (12 vs. 0%), and fatigue (35 vs. 21%) were reported more frequently in the SARS-CoV-2 group, although not statistically significant.

### Persistent complaints after the acute viral infection

None of the controls, but 27 children and adolescents from the SARS-CoV-2 group (33%) reported at least one persistent complaint (Table 1) at the time of study enrolment. Sixteen of these 27 children (59%) with a symptomatic SARS-CoV-2 infection reported at least one persisting symptom compared to 23.4% from the asymptomatic group (*P* < 0.05), resulting in a 2.3-fold higher probability of persistent complaints after a SARS-CoV-2 infection (CI 1.07–7.11). Nearly half of them reported decreased physical capacity, whereas ∼70% described having any neurological symptoms (Table 1). Except for pain, all other symptoms occurred significantly more often in subjects with symptomatic SARS-CoV-2 infection than in those with an asymptomatic course.

Neither patients nor controls showed reflex deficits or paresis in the clinical examination. In 72 participants of the SARS-CoV-2 group, lung function was additionally assessed, and 13 (18%) of them presented with abnormal findings (symptomatic SARS-CoV-2 infection: *n* = 6, reported persistent complaints: *n* = 5). Lung function was assessed in 33 participants in the control group, and 24% of them showed abnormal findings (n.s.) (4).

### Sensory profiles of quantitative sensory testing

**FIGURE 2:**
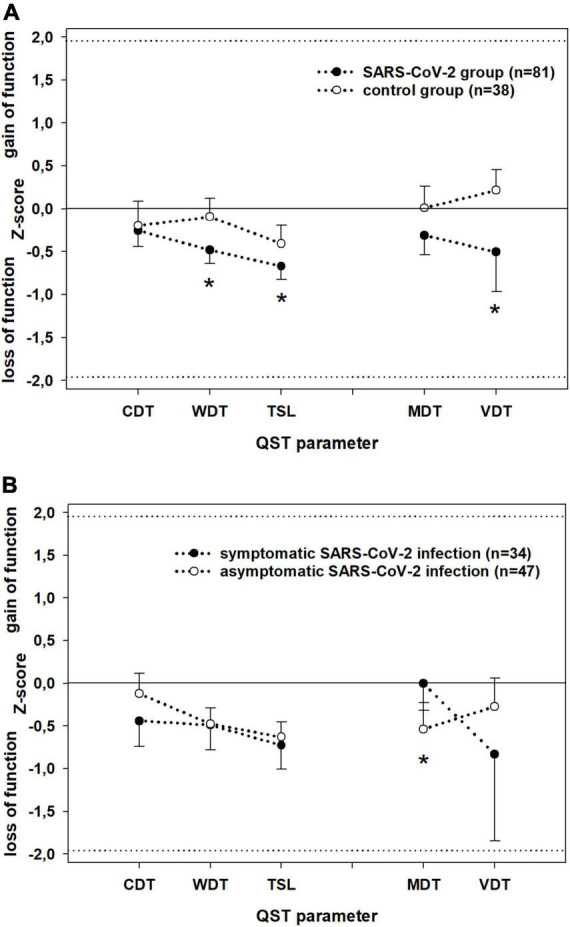
Detection thresholds at dorsal feet (z-score; mean ± SD) assessed by Quantitative Sensory Testing in **(A)** patients (black) vs. controls (white) and **(B)** patients with symptomatic (black) vs. patients with asymptomatic (white) SARS-CoV-2 infection. Parameters assessing the small (WDT; CDT; TSL) and large (VDT, MDT) fibre function have been separately linked by dotted lines for better visualisation. *Z*-values between –1.96 and 1.96: normal range of healthy subjects. *Z*-values > 0: gain of sensory function, *z*-values < 0: loss of sensory function. CDT, cold detection threshold; WDT, warm detection threshold; TSL, thermal sensory limen; MDT, mechanical detection threshold; VDT, vibration detection threshold. Significant differences (*P* < 0.05) are marked with *.

The somatosensory parameters assessed during QST were, on average, within the normal range in both patients and controls (Figure 2). However, the detection thresholds for thermal and mechanical stimuli were higher in the SARS-CoV-2 group than in the controls, with significant differences in WDT (*P* = 0.003), TSL (*P* = 0.02), and VDT (*P* = 0.02) (Figure 2). The SARS-CoV-2 group deviated significantly from an ideal control group (mean ± SD: 0 ± 1 *z*-values) in each QST parameter, whereas the control group deviated significantly from the ideal only in TSL (*p* = 0.012). Among those in the SARS-CoV-2 group who had asymptomatic infection, all QST parameters were significantly decreased, except CDT, and among those with a symptomatic SARS-CoV-2 infection, all QST parameters significantly decreased except MDT.

**FIGURE 3:**
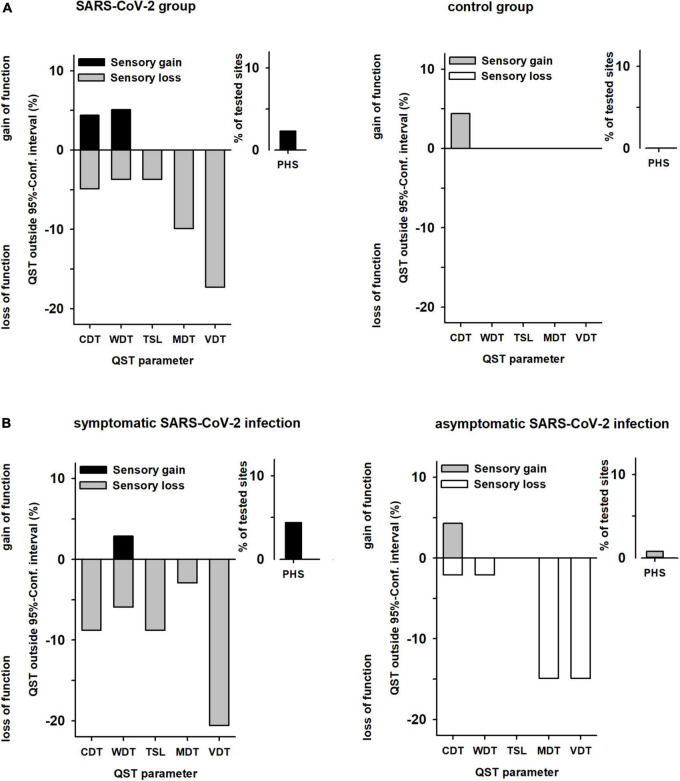
Abnormal values of sensory parameters in **(A)** patients vs. controls and **(B)** patients with symptomatic vs. patients with asymptomatic SARS-CoV-2 infection. QST values outside the 95% confidence interval (95% CI) of the reference data base (13). The *y*-axis shows percentage of subjects, with positive sensory signs mapped upward (gain of sensory function) and negative sensory signs mapped downward (loss of sensory function). **(A)** Patients *n* = 82, Controls *n* = 38. **(B)** Patients with symptomatic SARS-CoV-2 *n* = 35, Patients with asymptomatic SARS-CoV-2 *n* = 47. Absence of paradoxical heat sensations (PHS) is normal, so there are no negative signs for PHS. QST, quantitative sensory testing; CDT, cold detection threshold; WDT, warm detection threshold; TSL, thermal sensory limen; MDT, mechanical detection threshold; VDT, vibration detection threshold; PHS, paradoxical heat sensations.

There were no cases with abnormal sensory function (outside the reference values) in the control group. In contrast, in 36% of patients after SARS-CoV-2 infection, there was at least one abnormal QST parameter (*p* < 0.001). Abnormal values were found for thermal detection (WDT in 4%, CDT in 5%, and PHS in 6%) and even more frequently for mechanical detection (MDT in 10% and VDT in 17%; Figure 3A). Twenty-four patients (30%) had an abnormal result in only one QST parameter, five patients (6%) had ≥2 abnormal results. Sensory loss was observed in small fibre function in ten patients (12%) and in large fibre function in 21 patients (26%).

Patients with a history of symptomatic or asymptomatic SARS-CoV-2 infection had similar frequencies of at least one abnormally increased detection threshold (34 vs. 40%). However, within the subgroup with previous symptomatic SARS-CoV-2 infection a dysfunction of the small nerve fibres occurred significantly more frequently (21 vs. 4%; *P* < 0.05; Figure 3B).

### Relation between somatosensory dysfunction and subjective complaints

**FIGURE 4:**
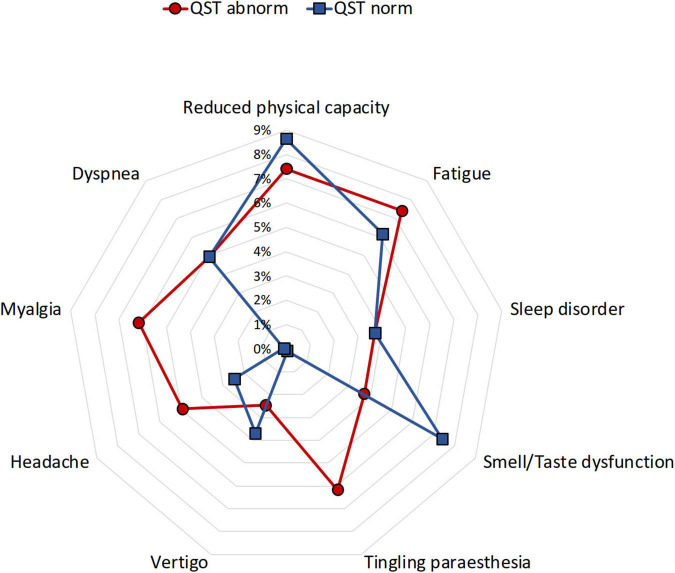
Distribution of frequent post-COVID complaints in children and adolescents with and without abnormal QST. QST, quantitative sensory testing. Normal QST (blue), abnormal QST (red).

Children and adolescents with persistent neurological complaints, particularly myalgia or paraesthesia, demonstrated more frequently abnormal increased somatosensory detection thresholds and paradoxical heat sensations (myalgia/paraesthesia 17 vs. 0%) (Table 1 and Figure 4). However, the occurrence of persistent long-COVID complaints was not associated with loss of sensory function (OR 1.3; CI 0.5–3.36).

Children with and without symptomatic infection had a similar prevalence of loss of sensory function (OR 1.29; CI 0.52–3.2).

The frequency of abnormal QST was similar in children with or without impaired lung function; of 13 out of 69 patients with SARS-CoV-2 infection and reduced lung function, five (39%) also had at least one abnormal detection threshold. Of the remaining 56 patients without abnormal lung parameters, 20 had an abnormal QST result (36%; *P* = 0.85).

A second follow-up QST assessment was performed in 26 patients of the SARS-CoV-2 group 4–12 months after infection (mean of 7.4 ± 2.4 months). The frequency of at least one abnormal QST parameter declined from 8 to 2 cases. Large fibre affection was recovered in all cases, whereas small fibre abnormalities persisted in two children, although only one of them reported ongoing complaints (leg pain and paraesthesia).

## Discussion

To the best of our knowledge, this is the first study showing that one-third of children and adolescents experience abnormal somatosensory function for up to 7 months after asymptomatic or mild symptomatic SARS-CoV-2 infection in a tertiary Corona out-patient clinic. In particular, patients with persistent neurological complaints, such as myalgia or paraesthesia, also presented with isolated somatosensory abnormalities without signs of lesions in the motor system (e.g., paresis or diminished tendon reflex during the clinical examination). No child in the control group, even those with a previous viral infection, reported any complaint 3 months later or showed abnormal values in the clinical or QST examination. Somatosensory profiles of the group with a history of a SARS-CoV-2 infection revealed on a group level a significant sensory loss for thermal stimuli, especially warmth, abnormalities in the perception of temperature changes, and sensory loss for vibration stimuli. At the individual level, at least one QST parameter was abnormal in one-third of the patients (*P* < 0.01), mostly related to large fibre function with complete recovery 7 months after infection. A loss of small fibre function was less frequent (11%) and was still present 4 months later.

The spectrum of persistent complaints 3 months after infection with SARS-CoV-2 corresponds to previous reports (3, 33). In our investigation, patients who reported myalgia or paraesthesia [with a prevalence of 7%, previous reports ranging from approximately 3 to 60% (3)] more often presented with a loss of sensory function. Sensory loss is known to occur in nerve damage of other aetiologies, on the one hand, in pathophysiologically well-characterised entities such as diabetic peripheral neuropathy and chemotherapy-induced neuropathy, and on the other hand, in multifactorial syndromes like fibromyalgia (18, 25, 27, 34–36).

The somatosensory assessment (QST) used here is a functional test with high sensitivity for the detection of neuropathy (17, 18, 23, 34, 37), due to a large norm database and using a rigorous protocol according to the DFNS (23, 29). Thus, only small deviations from normal values can be detected for the perception performance of different mechanical and thermal stimuli, independent of sex, age, and area, respectively (17, 18, 23, 24, 28, 38). QST has been recently recommended, e.g., for the assessment of sensory small fibre neuropathies (22). As it is a non-invasive and almost risk-free method, QST is a suitable tool for paediatric research, that has been used to describe the age- and sex-dependent development of somatosensory perception in healthy children (24) and evaluate somatosensory function in children and adolescents, e.g., with type I diabetes mellitus (25). In this entity, tactile hypoesthesia predicted abnormal findings in nerve conduction studies with high specificity and sensitivity, even in children without symptoms of manifest neuropathy, indicating that children have a greater ability to compensate for peripheral sensory loss of function. Our findings of abnormal somatosensory function in patients without complaints correspond to the findings in childhood diabetic neuropathy (25).

Although nerve conduction studies and skin biopsy, the latter assessing epidermal nerve fibre density in suspected small fibre neuropathy, remains the gold standard for diagnosing a neuropathy (39), these methods are not easily feasible in children without strong clinical indications. Hitherto, there are no data available in children with persisting complaints after SARS-CoV-2 infection. However, our results are in line with a recently published observational study in adults using corneal confocal microscopy, which reported morphological aberrations of the corneal innervation, that is, loss of small fibres and increased dendritic cells, the latter indicating immune-mediated pathomechanisms (40). In first small case series of adult long-COVID patients with new-onset paraesthesia, reduced intraepidermal nerve fibre densities were also detected in skin biopsies (41, 42).

It is undisputed, although less common in children than in adults, that SARS-CoV-2 can lead to severe neurological complications involving the central and autonomic nervous systems (43–46). Like other neurotropic viruses (Epstein-Barr virus, herpes, and influenza viruses) (47), corona virus has been reported to cause serious central nervous system disorders such as acute disseminated encephalomyelitis or myelitis in children (5, 6). An immune-mediated peripheral nerve dysfunction due to SARS-CoV-2 infection has been described only in case reports of Guillain-Barré syndrome and its variants with acute axonal neuropathy or cranial nerve affections or in children after paediatric inflammatory multisystem syndrome, where in 40% of cases, an unspecified involvement of the peripheral nervous system has been described (5, 6). Polyneuropathy syndromes have been previously described after influenza or EBV infection (9, 10). In adults, a significantly higher frequency of EBV reactivation has been recently reported in patients with long-COVID syndrome than in controls without persisting complaints after SARS-CoV-2 infection (48). Our findings suggest that somatosensory dysfunction is a common sequela of acute SARS-CoV-2 infection. It is presumably more frequent after symptomatic infections and may explain some of the most frequent reversible complaints, such as reduced physical performance, better than rare long-term pulmonary sequelae (4).

### Limitations

One limitation of our study is a probable recruitment bias, as those families whose children complained of persistent symptoms after infection were more likely to visit our special outpatient clinic and report for participation. Thus, our results cannot be used to estimate the prevalence of neurological dysfunction after SARS-CoV-2 infection. Moreover, the control group was heterogeneous, and details about the previous infections were not available in some subjects, apart from SARS-CoV-2 being excluded. In contrast to two recently reported larger healthy control cohorts, none of our controls complained of reduced physical activity, fatigue, and headache as possible post-pandemic symptoms (2, 49). Furthermore, the detection thresholds of the control group were within the normal range in contrast to the SARS-CoV-2 group. Therefore, the chance of false-positive QST findings in the group with a history of SARS-CoV-2 infection seems to be quite low. Due to the lack of additional assessment in our cohort, the origin of the impairment of sensory function cannot be specified. Further studies should include electrophysiological examination, morphological investigations of the nerve fibre density, for example by CCM, as well as profiles of antibodies against neural structures in serum and/or cerebrospinal fluid, which could possibly further elucidate the functional somatosensory abnormalities in the present study. Nevertheless, our data contribute to a better understanding of the effects of SARS-CoV-2 on the somatosensory system.

## Conclusion

This study demonstrates that loss of somatosensory function for both small and large nerve fibres and the corresponding pathways are detectable approximately 3 months after mild symptomatic and asymptomatic SARS-CoV-2 infection. Somatosensory abnormalities seem to occur independently of the severity of acute infection and mostly remain unnoticed. However, our findings indicate that some persistent symptoms in children and adolescents with long-COVID syndrome may have a somatic correlate. Therefore, in long-lasting or disabling cases with particular complaints such as myalgia or paraesthesia, nerve function should be considered as part of the diagnostic work-up after SARS-CoV-2 infection. Fortunately, the somatosensory changes seem to be reversible spontaneously over time in the majority of cases.

## Data availability statement

The original data presented in this study are included in the article/Supplementary material, further inquiries can be directed to the corresponding author.

## Ethics statement

The studies involving human participants were reviewed and approved by the Ethics Committee of the Ruhr University Bochum (Reg. no. 20-6927_1). Written informed consent to participate in this study was provided by the participants’ legal guardian/next of kin.

## Author contributions

LE, CM, FB, EE-K, and TL: conceptualisation. LE, CM, EE-K, and TL: methodology. LE, EE-K, and CM: formal analysis. LE, LK, and AS: investigation. LE: writing – original draft preparation. EE-K, CM, FB, AS, and TL: writing – review and editing. EE-K and TL: supervision. FB and TL: project administration. All authors have read and agreed to the published version of the manuscript.
