# Supplementary material for: Somatosensory abnormalities after infection with SARS-CoV-2 – A prospective case-control study in children and adolescents
Source: Front Pediatr. 2022 Oct 3;10:977827. doi: 10.3389/fped.2022.977827 (PMC9574195; doi:10.3389/fped.2022.977827)
Supplement: Supplementary file 1 [file Table_2.PDF]

Supplementary Material

1 Supplementary Table 1

Supplementary Table 1: Epidemiological data and infection characteristics for the children and adolescents.

	**Post-SARS-CoV-2 group**			**Control-group**			
	All	Symptomatic SARS-CoV-2	Asymptomatic SARS-CoV-2	All	Symptomatic infection within the last 6 months	No known infection within the last 6 months	OR (95% CI)
**Clinical data, No.**	**81**	**34**	**47**	**38**	**15**	**23**	-
Female, No. (%)	44 (54)	19 (56)	25 (53)	26 (68)	13 (87)	13 (57)	0.53 (0.24 - 1.2)
Male, No. (%)	37 (46)	15 (44)	22 (47)	12 (32)	2 (13)	10 (44)	-
Age, years, mean (SD)	11.3 (3.5)	11.1 (3.5)	11.5 (3.4)	10.3 (3.4)	10.1 (3.4)	10.4 (3.4)	-
Age group, 6-10 years, No. (%)	40 (49)	19 (56)	21 (45)	24 (63)	10 (67)	14 (61)	0.56 (0.25 - 1.22)
Age group, 11-18 years, No. (%)	41 (51)	15 (44)	26 (55)	14 (37)	5 (33)	9 (39)	1.8 (0.82 - 3.96)
BMI percentile by WHO growth chart <5	4 (5)	3 (9)	1 (2)	0 (0)	0 (0)	0 (0)	-
BMI percentile by WHO growth chart >85	22 (27)	8 (24)	14 (30)	6 (16)	4 (27)	2 (9)	-
Preexisting chronic diseases	10 (12)	8 (24)	2 (4)	8 (21)	4 (27)	4 (17)	-
**Any infection within 6 months prior assessment, No. (%)**	**39 (48)**	**34 (100)**	**4 (9)**	**15 (40)**	**15 (100)**	-	-
with hospitilization, No. (%)	5 (6)	5 (15)	-	0 (0)	0 (0)	-	-
with moderate infection, No. (%)	9 (11)	9 (27)	-	4 (11)	4 (27)	-	1.05 (0.3 - 3.64)
with non-severe infection, No. (%)	25 (31)	25 (74)	-	11 (29)	11 (73)	-	-
**Symptoms of acute infection***	**34 (42)**	**34 (100)**	-	**15 (40)**	**15 (100)**	-	-
Fever > 38.5°C, No. (%)	20 (25)	20 (59)	-	5 (13)	5 (33)	-	2.42 (0.84 - 6.98)
Cold symptoms, No. (%)	17 (21)	16 (47)	-	9 (24)	9 (60)	-	0.84 (0.34 - 2.11)
Sore throat, No. (%)	17 (21)	17 (50)	-	9 (24)	9 (60)	-	0.91 (0.36 - 2.26)
Cough, No. (%)	16 (20)	16 (47)	-	11 (29)	11 (73)	-	0.79 (0.33 - 1.88)
Dyspnea, No. (%)	8 (10)	8 (24)	-	2 (5)	2 (13)	-	1.95 (0.39 - 9.64)
Smell/Taste dysfunction, No. (%)	10 (12)	10 (30)	-	-	-	-	-
Headache, No. (%)	19 (24)	19 (56)	-	4 (11)	4 (27)	-	3.12 (0.99 - 9.8)
Limb pain, No. (%)	14 (17)	14 (41)	-	4 (11)	4 (27)	-	1.9 (0.59 - 6.18)
Fatigue, No. (%)	28 (35)	28 (82)	-	8 (21)	8 (53)	-	2.16 (0.88 - 5.32)
Diarrhea/Vomiting, No. (%)	11 (14)	11 (32)	-	2 (5)	2 (13)	-	2.79 (0.59 - 13.26)

*Multiple answers were possible. SD, standard deviation; OR, odds ratio; CI, confidence interval.
